# The Following Letters Are Extracted from the Independent Chronicle, Published at Boston, on the 15th Day of July, 1802

**Published:** 1802-12-01

**Authors:** Benj. Waterhouse

**Affiliations:** Cambridge


*The following Letters are extracted from the Independent Chronicle, published at Boston, on the* 15*th Day of July*, 1802.

MESSRS. EDITORS,

THE public muft have perceived, before this time, that I have never failed to examine every report which has appeared to militate againft my early and uniform affertion, *that the kine-pock afforded a sure, safe, and permanent security against the small-pox*; and, that againft every fuch report, I have invariably oppofed a public contradiction. But the origin of thefe falfe reports has not been confined to our own country; fome of them owe their origin to people in England, who have written them to their friends here. Thefe I have, in like manner, traced to their fource, and found them all turn out to the honour of my doctrine.

Of this nature is the one now offered to the public. It has been obferved in England, as well as in this country, that elderly people, fuch as Mr. E. have been flow to believe in the efficacy of the Jennerian difcovery. It is no wonder they are fceptical, having known, in the courfe of their lives, numberlefs whims that boaft their exiftence to-day, and to-morrow are no more.

Some perfons had written very difcouraging accounts of the kine-pock inoculation in England, to their friends here, and had mentioned names and circumftances, and quoted Mr. E. as their authority. Coming through fo refpectable a channel, it could not but make a difagreeable impreffion on fome, whofe families had received the diftemper from my hands. I therefore felt it a duty which I owed to them and to myfelf, to requeft further particulars from the gentleman quoted. My letter paffed through the hands of Mr. Ring, a furgeon of the firft rank in London; and who, next to Dr. Jenner, is the moft celebrated Vaccine Inoculator in Europe. Mr. Erving merits the thanks of his countrymen for this candid, explicit, and I may add, patriotic letter.

*Cambridge, July* 13, 1802. BENJ. WATERHOUSE.

---

*A Letter from* GEORGE ERVING, *Esq. of London, to Dr.* WATERHOUSE, *at Cambridge, in America.*

SIR,

I lately had the pleafure of receiving your letter of the 12th of February, through your friend, Mr. Ring. It gives me much much concern to think, that any thing which might inadvertently have fallen from me, refpecting the Vaccine Inoculation, fhould have created any alarm or apprehenfion in any amongft you, who have taken, or might be difpofed to take, this difeafe; or in the leaft to have abated your happy progrefs in differminating this diforder throughout the United States, and thereby exterminating that moft inveterate enemy of mankind, the Small-pox. The Vaccine Inoculation, fo lately brought into ufe, which is daily extending, and gaining frefh credit as it extends, has had the fate of all new difcoveries, to contend with a variety of oppofition. All innovations from our common habits of thinking and acting, muft neceffarily meet with opponents; and this oppofition, or collifion of opinions, acting according to the importance of the object, begets enquiry, inveftigation, and experiments, till at length truth is fairly brought out and eftablifhed, or error detected and exploded. At the firft introduction of the Vaccine Inoculation, there was a great deal to learn, which experiment and practice alone could teach; and there can be no wonder, in the early ftages of the practice, where in many cafes there were different appearances and refults, if men of the moft enlightened minds fhould differ in opinion as to its operation and effects; particularly that one effect of totally difcharging the habit from all fufceptibility of the variolous infection, in which confifts its whole ultimate value; and this could be determined by time alone. Some Phyficians, I underftand, have fuppofed and acted upon this fuppofition, that this difeafe was only a different fpecies of fmall-pox; and that the patient may receive them both together with the beft effect. But this doctrine has been exploded: perfeverance in the practice had led to new difcoveries and improvements, which have produced the happy confequence of filencing oppofition; and forcing conviction upon every candid mind. I am no Phyfician; nor has it fallen in my way to converfe much with gentlemen of the profeffion on the fubject; yet, as this is a difcovery of fuch happy promife to mankind, I have felt myfelf more than commonly interefted in its fate. I have therefore read books, made enquiries, and heard opinions; and on making up my judgment on the cafe, I confefs myfelf to have been on the fceptical fide of the queftion. But the evidences of facts have latterly become fo ftrong and irrefiftible, as to have borne away all my objections; and I have no hefitation even to declare myfelf an entire convert, and to difmifs all my former doubts. I had often heard and believed, that this diforder left a foulnefs in the blood, efpecially in children and infants, which required a confiderable time to eradicate; and this, I underftand, has not been been without example in the early ſtages of the practice. I alſo thought that the time was much too ſhort to pronounce decidedly on its permanent effects; but from ſome particular caſes adduced in your friend, Mr. Ring's valuable book, I with great ſatisfaction renounce this opinion. I now can eaſily believe, if any ill humour ſhould remain in the blood after the diſeaſe has paſſed, or any ſuſceptibility in the habit to receive the variolous infection, it muſt be owing ſolely to the wrong ſort, or the impure or vitiated ſtate of the matter with which the patient was inoculated; and this ſeems to be pretty well aſcertained by all the practitioners in this diſeaſe. As to what Dr. S——— ſaid, it was, as well as I can recollect, that two young gentlemen of Oxford, (whether they were young men or young children is no way material as to the fact) had been inoculated for the Vaccine Pox by Dr. Jenner, which they got well through; and that a few months, or a conſiderable time after, they were both taken ill of the Small-pox in the natural way, of which one of them died. He mentioned no particulars, as I dare ſay he knew none, of the kind of matter, or the quality of it, with which they were inoculated. It is poſſible that the vaccine virus, by expoſure to the air, or ſome other cauſe, had undergone a change, ſo as to have altered or wholly deſtroyed its effects: and that this may have been the caſe is pretty evident from later diſcoveries and improvements in the practice, as your ingenious friends, Dr. Jenner and Mr. Ring, may more clearly explain to you.

Great merit is due to Dr. Jenner, in the firſt inſtance, and to thoſe able and worthy practitioners who have followed and ſupported him, in drawing from obſcurity this long-neglected diſeaſe; and have, with ſuch unremitted labour and ingenuity, ſtruggled againſt every ſpecies of oppoſition, till they have brought conviction home to every candid mind. Much praiſe and gratitude are due to you, Sir, from your country, for the lead which you have taken in this infant ſcience. May increaſed ſucceſs attend you in your progreſs; and may ample rewards compenſate you for all your labours.

I am, with much reſpect, SIR,

Your moſt obedient ſervant,

GEO. IRVING.

*Hanover Square, London, April* 20, 1802.

---

*** It is a duty we owe to Dr. JENNER, and to the cauſe of Truth, to aſſert, that Dr. Jenner has never inoculated any perſon at Oxford; conſequently, the whole report which occaſioned ſo much alarm in the minds of our Tranſatlantic brethren is totally deſtitute of foundation.

